# Diaqua­[5,5′-dicarb­oxy-2,2′-(ethane-1,2-di­yl)bis­(1*H*-imidazole-4-carboxyl­ato)]cobalt(II)

**DOI:** 10.1107/S1600536811019672

**Published:** 2011-05-28

**Authors:** Ying Wang, Xin-Lian Gao

**Affiliations:** a Department of Geriatrics, The First Affiliated Hospital, Zhengzhou University, Zhengzhou 450000, People’s Republic of China; b Henan University of Traditional Chinese Medicine, Zhengzhou 450008, People’s Republic of China

## Abstract

In the title complex, [Co(C_12_H_8_N_4_O_8_)(H_2_O)_2_], the Co^II^ atom is coordinated by two N and two O atoms of the tetra­dentate 5,5′-dicarb­oxy-2,2′-(ethane-1,2-di­yl)bis­(1*H*-imidazole-4-carboxy­l­ate) anion. The slightly distorted octa­hedral coordination environment is completed by the O atoms of two water mol­ecules in axial positions. An intra­molecular O—H⋯O hydrogen bond between the carb­oxy and carboxyl­ate groups stabilizes the mol­ecular configuration. Adjacent mol­ecules are linked through O—H⋯O and N—H⋯O hydrogen bonds between the carb­oxy/carboxyl­ate groups, water mol­ecules and imidazole fragments into a three-dimensional network.

## Related literature

For background to complexes based on 1*H*-imidazole-4,5-dicarb­oxy­lic acid and its derivatives, see: Das *et al.* (2010 ▶); Sun *et al.* (2010 ▶); Zhang *et al.* (2010 ▶).
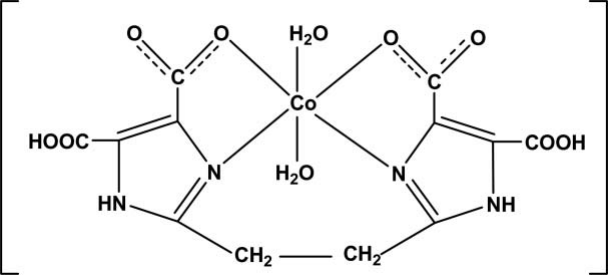


## Experimental

### 

#### Crystal data

[Co(C_12_H_8_N_4_O_8_)(H_2_O)_2_]
*M* *_r_* = 431.19
Orthorhombic, 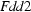

*a* = 24.683 (5) Å
*b* = 27.885 (6) Å
*c* = 8.7340 (17) Å
*V* = 6012 (2) Å^3^
*Z* = 16
Mo *K*α radiation
μ = 1.21 mm^−1^
*T* = 293 K
0.18 × 0.14 × 0.09 mm

#### Data collection

Rigaku Saturn CCD diffractometer
Absorption correction: multi-scan (*CrystalClear*; Rigaku/MSC, 2006 ▶) *T*_min_ = 0.811, *T*_max_ = 0.899
7187 measured reflections
2693 independent reflections
2223 reflections with *I* > 2σ(*I*)
*R*_int_ = 0.046

#### Refinement

*R*[*F* ^2^ > 2σ(*F* ^2^)] = 0.047
*wR*(*F* ^2^) = 0.079
*S* = 1.01
2693 reflections
245 parameters
1 restraint
H-atom parameters constrained
Δρ_max_ = 0.37 e Å^−3^
Δρ_min_ = −0.35 e Å^−3^
Absolute structure: Flack (1983) ▶, 1126 Friedel pairs
Flack parameter: 0.20 (2)

### 

Data collection: *CrystalClear* (Rigaku/MSC, 2006 ▶); cell refinement: *CrystalClear*; data reduction: *CrystalClear*; program(s) used to solve structure: *SHELXS97* (Sheldrick, 2008 ▶); program(s) used to refine structure: *SHELXL97* (Sheldrick, 2008 ▶); molecular graphics: *XP* in *SHELXTL* (Sheldrick, 2008 ▶); software used to prepare material for publication: *SHELXTL*.

## Supplementary Material

Crystal structure: contains datablocks global, I. DOI: 10.1107/S1600536811019672/wm2487sup1.cif

Structure factors: contains datablocks I. DOI: 10.1107/S1600536811019672/wm2487Isup2.hkl

Additional supplementary materials:  crystallographic information; 3D view; checkCIF report

## Figures and Tables

**Table 1:** Selected bond lengths (Å)

Co1—O10	2.043 (4)
Co1—N3	2.044 (4)
Co1—N1	2.048 (4)
Co1—O9	2.118 (4)
Co1—O1	2.153 (3)
Co1—O5	2.168 (4)

**Table 2:** Hydrogen-bond geometry (Å, °)

*D*—H⋯*A*	*D*—H	H⋯*A*	*D*⋯*A*	*D*—H⋯*A*
O3—H3⋯O2	0.85	1.65	2.461 (5)	157
O7—H7⋯O6	0.85	1.72	2.550 (5)	166
O10—H3*W*⋯O3^i^	0.85	2.18	2.799 (5)	129
N2—H2*A*⋯O6^ii^	0.86	2.16	2.904 (5)	145
N4—H4*A*⋯O5^iii^	0.86	2.04	2.878 (5)	166
O9—H1*W*⋯O4^iv^	0.85	1.89	2.730 (5)	170
O9—H2*W*⋯O7^v^	0.85	2.06	2.834 (5)	150
O10—H4*W*⋯O8^vi^	0.85	2.22	2.958 (5)	145

Symmetry codes: (i) 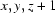; (ii) 
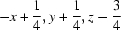
; (iii) 
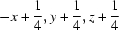
; (iv) 
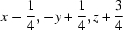
; (v) 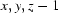; (vi) 
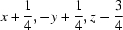
.

**Fig. 1.**
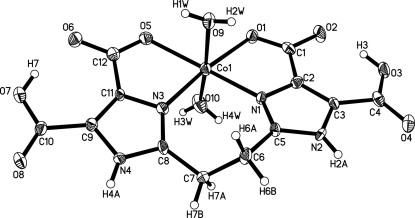
View of the molecular structure of the title complex, showing the labelling of the atoms. Displacement ellipsoids are drawn at the 30% probability level.

**Fig. 2.**
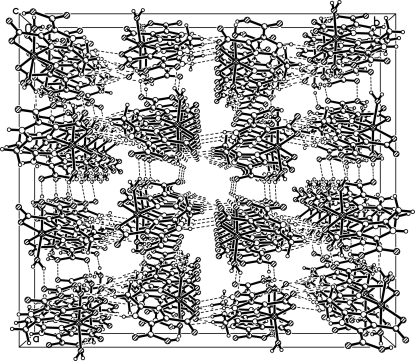
View of the crystal packing of the title complex, showing the three-dimensional structure stabilized by hydrogen bonds.

## supplementary crystallographic information

### Comment

A large number of metal complexes constructed from the ligand 1*H*-imidazole-4,5-dicarboxylic acid or its derivatives have been reported. This ligand shows versatile binding modes and high binding capacity with almost all soft and hard metal ions (Das *et al.*, 2010; Sun *et al.*, 2010; Zhang *et al.*, 2010). In order to further explore complexes with novel structures, we obtained the title complex [Co(H_4_eidc)(H_2_O)_2_], (I), through the reaction of 2,2'-(ethane-1,2-diyl)bis(1*H*-imidazole-4,5-dicarboxylic acid (H_6_eidc) with cobalt dichloride.

As shown in Figure 1, the Co(II) cation in (I) is hexacoordinated and features a slightly octahedral coordination environment. N1, O1, N3, O5 atoms from the tetradentate H_4_eidc^2-^ anion coordinate to the cation in a chelating fashion and O9, O10 atoms from water molecules complete the coordination polyhedron. Atoms N1, N3, O1, O5 and Co are nearly co-planar (the mean deviation from the plane is 0.08 Å). The bond angle between the O atoms of the two water molecules and the metal is 171.87 (13) °. As shown in Figure 2, intramolecular O—H···O hydrogen bonds between the carboxyl/carboxylate groups stabilize the molecular configuration whereas O—H···O and N—H···O hydrogen bonds between the water molecules and carboxylate O atoms and between imidazole groups and carboxylate O atoms of adjacent molecules consolidate the crystal packing.

### Experimental

The ligand 2,2'-(ethane-1,2-diyl)bis(1*H*-imidazole-4,5-dicarboxylic acid (0.05 mmol) in methanol (4 ml) was added dropwise to a methanol solution (3 ml) of cobalt dichloride (0.05 mmol). The resulting solution was allowed to stand at room temperature. After four weeks red crystals with good quality were obtained from the filtrate and dried in air.

### Refinement

The crystal of the title complex was twinned (twin ratio 0.8:0.2). H atoms were positioned geometrically and refined as riding atoms, with C—H = 0.97 Å, N—H = 0.86 Å and O—H = 0.85 Å, and with U_iso_(H) = 1.2 U_eq_(C,N,O).

### Crystal data

**Table:**

[Co(C_12_H_8_N_4_O_8_)(H_2_O)_2_]	*F*(000) = 3504
*M**_r_* = 431.19	*D*_x_ = 1.906 Mg m^−^^3^
Orthorhombic, *F**d**d*2	Mo *K*α radiation, λ = 0.71073 Å
Hall symbol: F 2 -2d	θ = 2.6–27.8°
*a* = 24.683 (5) Å	µ = 1.21 mm^−^^1^
*b* = 27.885 (6) Å	*T* = 293 K
*c* = 8.7340 (17) Å	Prism, red
*V* = 6012 (2) Å^3^	0.18 × 0.14 × 0.09 mm
*Z* = 16	

### Data collection

**Table:**

Rigaku Saturn CCD diffractometer	2693 independent reflections
Radiation source: fine-focus sealed tube	2223 reflections with *I* > 2σ(*I*)
graphite	*R*_int_ = 0.046
Detector resolution: 28.5714 pixels mm^-1^	θ_max_ = 26.0°, θ_min_ = 2.2°
ω scans	*h* = −30→11
Absorption correction: multi-scan (*CrystalClear*; Rigaku/MSC, 2006)	*k* = −31→33
*T*_min_ = 0.811, *T*_max_ = 0.899	*l* = −10→10
7187 measured reflections	

### Refinement

**Table:**

Refinement on *F*^2^	Secondary atom site location: difference Fourier map
Least-squares matrix: full	Hydrogen site location: inferred from neighbouring sites
*R*[*F*^2^ > 2σ(*F*^2^)] = 0.047	H-atom parameters constrained
*wR*(*F*^2^) = 0.079	*w* = 1/[σ^2^(*F*_o_^2^) + (0.0302*P*)^2^] where *P* = (*F*_o_^2^ + 2*F*_c_^2^)/3
*S* = 1.01	(Δ/σ)_max_ < 0.001
2693 reflections	Δρ_max_ = 0.37 e Å^−^^3^
245 parameters	Δρ_min_ = −0.35 e Å^−^^3^
1 restraint	Absolute structure: Flack (1983), 1126 Friedel pairs
Primary atom site location: structure-invariant direct methods	Flack parameter: 0.20 (2)

### Special details

**Table:**

Geometry. All e.s.d.'s (except the e.s.d. in the dihedral angle between two l.s. planes) are estimated using the full covariance matrix. The cell e.s.d.'s are taken into account individually in the estimation of e.s.d.'s in distances, angles and torsion angles; correlations between e.s.d.'s in cell parameters are only used when they are defined by crystal symmetry. An approximate (isotropic) treatment of cell e.s.d.'s is used for estimating e.s.d.'s involving l.s. planes.
Refinement. Refinement of *F*^2^ against ALL reflections. The weighted *R*-factor *wR* and goodness of fit *S* are based on *F*^2^, conventional *R*-factors *R* are based on *F*, with *F* set to zero for negative *F*^2^. The threshold expression of *F*^2^ > σ(*F*^2^) is used only for calculating *R*-factors(gt) *etc*. and is not relevant to the choice of reflections for refinement. *R*-factors based on *F*^2^ are statistically about twice as large as those based on *F*, and *R*- factors based on ALL data will be even larger.

### Fractional atomic coordinates and isotropic or equivalent isotropic displacement parameters (Å^2^)

**Table:**

	*x*	*y*	*z*	*U*_iso_*/*U*_eq_	
Co1	0.15380 (3)	0.073588 (18)	0.83795 (11)	0.02698 (17)	
N1	0.17562 (18)	0.12247 (13)	0.6742 (5)	0.0234 (9)	
N2	0.19660 (17)	0.17992 (13)	0.5122 (5)	0.0266 (11)	
H2A	0.2014	0.2082	0.4751	0.032*	
N3	0.13408 (17)	0.12246 (13)	1.0033 (5)	0.0246 (10)	
N4	0.11839 (17)	0.17995 (13)	1.1637 (5)	0.0238 (11)	
H4A	0.1174	0.2081	1.2039	0.029*	
O1	0.18202 (16)	0.02761 (10)	0.6567 (4)	0.0338 (9)	
O2	0.21834 (16)	0.02922 (12)	0.4193 (4)	0.0447 (11)	
O3	0.24378 (15)	0.09236 (12)	0.2360 (4)	0.0375 (9)	
H3	0.2268	0.0720	0.2902	0.045*	
O4	0.24502 (15)	0.17202 (12)	0.2168 (4)	0.0381 (9)	
O5	0.11949 (15)	0.02774 (11)	1.0134 (4)	0.0329 (9)	
O6	0.07457 (15)	0.03203 (11)	1.2358 (4)	0.0366 (10)	
O7	0.05925 (15)	0.09672 (12)	1.4375 (4)	0.0377 (10)	
H7	0.0609	0.0725	1.3788	0.045*	
O8	0.06967 (16)	0.17524 (12)	1.4593 (5)	0.0422 (10)	
O9	0.07403 (15)	0.07148 (10)	0.7490 (4)	0.0338 (9)	
H1W	0.0467	0.0730	0.8078	0.041*	
H2W	0.0669	0.0888	0.6714	0.041*	
O10	0.22935 (16)	0.06549 (14)	0.9300 (5)	0.0521 (12)	
H3W	0.2464	0.0581	1.0112	0.062*	
H4W	0.2547	0.0804	0.8846	0.062*	
C1	0.1996 (2)	0.04938 (18)	0.5417 (6)	0.0318 (13)	
C2	0.1972 (2)	0.10239 (16)	0.5440 (6)	0.0263 (12)	
C3	0.2095 (2)	0.13797 (16)	0.4409 (5)	0.0229 (11)	
C4	0.2348 (2)	0.13487 (19)	0.2884 (6)	0.0317 (13)	
C5	0.1748 (2)	0.16963 (16)	0.6526 (6)	0.0236 (12)	
C6	0.1512 (2)	0.20512 (18)	0.7602 (6)	0.0331 (14)	
H6A	0.1122	0.2012	0.7587	0.040*	
H6B	0.1590	0.2369	0.7208	0.040*	
C7	0.1698 (2)	0.20335 (17)	0.9274 (6)	0.0300 (13)	
H7A	0.2079	0.1949	0.9295	0.036*	
H7B	0.1664	0.2352	0.9708	0.036*	
C8	0.1398 (2)	0.16921 (17)	1.0263 (6)	0.0257 (12)	
C9	0.09834 (19)	0.13883 (16)	1.2301 (6)	0.0244 (11)	
C10	0.0749 (2)	0.13908 (18)	1.3862 (6)	0.0278 (12)	
C11	0.1094 (2)	0.10327 (15)	1.1278 (5)	0.0216 (12)	
C12	0.1005 (2)	0.05041 (17)	1.1266 (6)	0.0291 (14)	

### Atomic displacement parameters (Å^2^)

**Table:**

	*U*^11^	*U*^22^	*U*^33^	*U*^12^	*U*^13^	*U*^23^
Co1	0.0348 (4)	0.0239 (3)	0.0222 (3)	0.0011 (4)	0.0053 (3)	−0.0014 (3)
N1	0.031 (2)	0.020 (2)	0.019 (2)	0.0002 (18)	0.0058 (19)	0.0023 (17)
N2	0.031 (3)	0.025 (2)	0.024 (3)	−0.0094 (19)	0.008 (2)	0.005 (2)
N3	0.032 (3)	0.021 (2)	0.022 (2)	0.0027 (18)	−0.001 (2)	0.0001 (19)
N4	0.032 (3)	0.018 (2)	0.022 (3)	−0.0025 (18)	0.000 (2)	0.0005 (18)
O1	0.050 (3)	0.0237 (18)	0.027 (2)	−0.0017 (17)	0.0098 (19)	−0.0023 (17)
O2	0.067 (3)	0.039 (2)	0.028 (2)	0.0071 (19)	0.017 (2)	−0.0122 (18)
O3	0.044 (3)	0.048 (2)	0.021 (2)	0.0008 (18)	0.0092 (18)	−0.0042 (18)
O4	0.032 (2)	0.053 (2)	0.029 (2)	0.0013 (18)	0.0013 (19)	0.0115 (19)
O5	0.044 (3)	0.0230 (18)	0.032 (2)	−0.0022 (17)	0.001 (2)	−0.0014 (18)
O6	0.050 (3)	0.0273 (18)	0.032 (2)	−0.0022 (17)	0.0041 (19)	0.0078 (18)
O7	0.047 (3)	0.044 (2)	0.022 (2)	−0.0041 (19)	0.0046 (19)	−0.0017 (18)
O8	0.055 (3)	0.042 (2)	0.030 (2)	−0.0036 (18)	0.015 (2)	−0.0133 (19)
O9	0.032 (2)	0.0422 (19)	0.028 (2)	0.0034 (16)	0.0054 (17)	0.0032 (19)
O10	0.045 (3)	0.077 (3)	0.034 (3)	0.012 (2)	−0.005 (2)	−0.006 (2)
C1	0.035 (3)	0.034 (3)	0.027 (3)	0.001 (2)	−0.002 (2)	−0.011 (3)
C2	0.024 (3)	0.025 (3)	0.029 (3)	−0.001 (2)	0.004 (2)	−0.001 (2)
C3	0.025 (3)	0.028 (3)	0.016 (3)	0.000 (2)	0.001 (2)	−0.003 (2)
C4	0.027 (3)	0.046 (3)	0.023 (3)	0.006 (3)	−0.005 (2)	−0.002 (3)
C5	0.026 (3)	0.026 (3)	0.018 (3)	0.004 (2)	0.006 (2)	0.000 (2)
C6	0.044 (4)	0.025 (3)	0.030 (3)	0.006 (2)	0.016 (3)	0.003 (2)
C7	0.041 (4)	0.023 (3)	0.025 (3)	−0.008 (2)	0.004 (3)	0.000 (2)
C8	0.037 (3)	0.026 (3)	0.014 (3)	0.005 (2)	0.001 (2)	0.002 (2)
C9	0.030 (3)	0.023 (3)	0.020 (3)	0.001 (2)	−0.002 (2)	0.005 (2)
C10	0.023 (3)	0.035 (3)	0.025 (3)	−0.002 (2)	0.004 (2)	0.001 (3)
C11	0.032 (3)	0.019 (2)	0.014 (3)	0.000 (2)	0.001 (2)	0.004 (2)
C12	0.030 (3)	0.031 (3)	0.027 (4)	0.002 (2)	−0.002 (3)	0.002 (3)

### Geometric parameters (Å, °)

**Table:**

Co1—O10	2.043 (4)	O6—C12	1.258 (6)
Co1—N3	2.044 (4)	O7—C10	1.321 (6)
Co1—N1	2.048 (4)	O7—H7	0.8501
Co1—O9	2.118 (4)	O8—C10	1.200 (5)
Co1—O1	2.153 (3)	O9—H1W	0.8500
Co1—O5	2.168 (4)	O9—H2W	0.8498
N1—C5	1.329 (6)	O10—H3W	0.8501
N1—C2	1.375 (6)	O10—H4W	0.8499
N2—C3	1.363 (6)	C1—C2	1.479 (7)
N2—C5	1.370 (6)	C2—C3	1.374 (6)
N2—H2A	0.8600	C3—C4	1.474 (7)
N3—C8	1.327 (6)	C5—C6	1.484 (7)
N3—C11	1.357 (6)	C6—C7	1.532 (6)
N4—C8	1.345 (6)	C6—H6A	0.9700
N4—C9	1.377 (6)	C6—H6B	0.9700
N4—H4A	0.8600	C7—C8	1.484 (7)
O1—C1	1.252 (6)	C7—H7A	0.9700
O2—C1	1.293 (6)	C7—H7B	0.9700
O3—C4	1.290 (6)	C9—C11	1.362 (6)
O3—H3	0.8501	C9—C10	1.481 (7)
O4—C4	1.236 (6)	C11—C12	1.490 (6)
O5—C12	1.264 (6)		
			
O10—Co1—N3	90.75 (16)	C3—C2—N1	109.5 (4)
O10—Co1—N1	96.21 (17)	C3—C2—C1	134.7 (5)
N3—Co1—N1	96.43 (12)	N1—C2—C1	115.7 (4)
O10—Co1—O9	171.87 (13)	N2—C3—C2	105.6 (4)
N3—Co1—O9	93.23 (14)	N2—C3—C4	124.2 (4)
N1—Co1—O9	90.39 (15)	C2—C3—C4	130.1 (5)
O10—Co1—O1	85.88 (15)	O4—C4—O3	123.8 (5)
N3—Co1—O1	173.42 (16)	O4—C4—C3	119.7 (5)
N1—Co1—O1	78.35 (15)	O3—C4—C3	116.6 (5)
O9—Co1—O1	90.83 (14)	N1—C5—N2	109.2 (4)
O10—Co1—O5	90.76 (16)	N1—C5—C6	125.2 (5)
N3—Co1—O5	78.52 (15)	N2—C5—C6	125.5 (4)
N1—Co1—O5	171.47 (16)	C5—C6—C7	117.7 (5)
O9—Co1—O5	83.09 (13)	C5—C6—H6A	107.9
O1—Co1—O5	107.14 (10)	C7—C6—H6A	107.9
C5—N1—C2	106.9 (4)	C5—C6—H6B	107.9
C5—N1—Co1	139.0 (4)	C7—C6—H6B	107.9
C2—N1—Co1	114.1 (3)	H6A—C6—H6B	107.2
C3—N2—C5	108.7 (4)	C8—C7—C6	115.2 (5)
C3—N2—H2A	125.6	C8—C7—H7A	108.5
C5—N2—H2A	125.6	C6—C7—H7A	108.5
C8—N3—C11	108.3 (4)	C8—C7—H7B	108.5
C8—N3—Co1	137.5 (4)	C6—C7—H7B	108.5
C11—N3—Co1	114.2 (3)	H7A—C7—H7B	107.5
C8—N4—C9	109.4 (4)	N3—C8—N4	108.2 (4)
C8—N4—H4A	125.3	N3—C8—C7	126.5 (5)
C9—N4—H4A	125.3	N4—C8—C7	125.0 (4)
C1—O1—Co1	114.4 (3)	C11—C9—N4	104.9 (4)
C4—O3—H3	109.4	C11—C9—C10	133.3 (5)
C12—O5—Co1	113.8 (3)	N4—C9—C10	121.6 (5)
C10—O7—H7	119.6	O8—C10—O7	122.7 (5)
Co1—O9—H1W	121.1	O8—C10—C9	122.4 (5)
Co1—O9—H2W	118.0	O7—C10—C9	115.0 (4)
H1W—O9—H2W	106.7	N3—C11—C9	109.2 (4)
Co1—O10—H3W	143.7	N3—C11—C12	116.8 (4)
Co1—O10—H4W	115.7	C9—C11—C12	134.0 (5)
H3W—O10—H4W	98.3	O6—C12—O5	125.3 (5)
O1—C1—O2	125.2 (5)	O6—C12—C11	118.2 (5)
O1—C1—C2	117.4 (4)	O5—C12—C11	116.5 (4)
O2—C1—C2	117.4 (5)		
			
O10—Co1—N1—C5	−98.6 (6)	C2—C3—C4—O4	−176.2 (5)
N3—Co1—N1—C5	−7.2 (6)	N2—C3—C4—O3	−179.1 (4)
O9—Co1—N1—C5	86.1 (5)	C2—C3—C4—O3	5.5 (8)
O1—Co1—N1—C5	176.9 (6)	C2—N1—C5—N2	−1.4 (6)
O10—Co1—N1—C2	82.6 (3)	Co1—N1—C5—N2	179.8 (4)
N3—Co1—N1—C2	174.1 (3)	C2—N1—C5—C6	175.6 (5)
O9—Co1—N1—C2	−92.6 (3)	Co1—N1—C5—C6	−3.3 (9)
O1—Co1—N1—C2	−1.9 (3)	C3—N2—C5—N1	2.5 (6)
O10—Co1—N3—C8	85.1 (6)	C3—N2—C5—C6	−174.4 (5)
N1—Co1—N3—C8	−11.2 (6)	N1—C5—C6—C7	52.6 (8)
O9—Co1—N3—C8	−102.0 (5)	N2—C5—C6—C7	−131.0 (5)
O5—Co1—N3—C8	175.7 (6)	C5—C6—C7—C8	−85.6 (5)
O10—Co1—N3—C11	−92.9 (4)	C11—N3—C8—N4	−0.7 (6)
N1—Co1—N3—C11	170.8 (3)	Co1—N3—C8—N4	−178.7 (4)
O9—Co1—N3—C11	80.0 (3)	C11—N3—C8—C7	173.0 (5)
O5—Co1—N3—C11	−2.2 (3)	Co1—N3—C8—C7	−5.0 (9)
O10—Co1—O1—C1	−95.3 (4)	C9—N4—C8—N3	−0.1 (6)
N1—Co1—O1—C1	2.0 (4)	C9—N4—C8—C7	−173.9 (5)
O9—Co1—O1—C1	92.2 (4)	C6—C7—C8—N3	56.8 (8)
O5—Co1—O1—C1	175.2 (3)	C6—C7—C8—N4	−130.5 (5)
O10—Co1—O5—C12	95.0 (4)	C8—N4—C9—C11	0.9 (5)
N3—Co1—O5—C12	4.4 (4)	C8—N4—C9—C10	176.7 (5)
O9—Co1—O5—C12	−90.3 (4)	C11—C9—C10—O8	178.3 (5)
O1—Co1—O5—C12	−179.1 (3)	N4—C9—C10—O8	3.8 (8)
Co1—O1—C1—O2	−179.7 (4)	C11—C9—C10—O7	−3.0 (8)
Co1—O1—C1—C2	−1.7 (6)	N4—C9—C10—O7	−177.4 (4)
C5—N1—C2—C3	−0.2 (6)	C8—N3—C11—C9	1.2 (6)
Co1—N1—C2—C3	178.9 (3)	Co1—N3—C11—C9	179.8 (3)
C5—N1—C2—C1	−177.5 (4)	C8—N3—C11—C12	−178.4 (4)
Co1—N1—C2—C1	1.7 (5)	Co1—N3—C11—C12	0.2 (5)
O1—C1—C2—C3	−176.3 (6)	N4—C9—C11—N3	−1.3 (5)
O2—C1—C2—C3	1.9 (9)	C10—C9—C11—N3	−176.4 (5)
O1—C1—C2—N1	0.1 (7)	N4—C9—C11—C12	178.2 (5)
O2—C1—C2—N1	178.3 (5)	C10—C9—C11—C12	3.1 (10)
C5—N2—C3—C2	−2.5 (6)	Co1—O5—C12—O6	173.6 (4)
C5—N2—C3—C4	−178.9 (5)	Co1—O5—C12—C11	−5.6 (6)
N1—C2—C3—N2	1.7 (6)	N3—C11—C12—O6	−175.4 (5)
C1—C2—C3—N2	178.3 (5)	C9—C11—C12—O6	5.1 (8)
N1—C2—C3—C4	177.8 (5)	N3—C11—C12—O5	3.8 (7)
C1—C2—C3—C4	−5.7 (10)	C9—C11—C12—O5	−175.6 (5)
N2—C3—C4—O4	−0.8 (8)		

### Hydrogen-bond geometry (Å, °)

**Table:**

*D*—H···*A*	*D*—H	H···*A*	*D*···*A*	*D*—H···*A*
O3—H3···O2	0.85	1.65	2.461 (5)	157
O7—H7···O6	0.85	1.72	2.550 (5)	166
O10—H3W···O3^i^	0.85	2.18	2.799 (5)	129
N2—H2A···O6^ii^	0.86	2.16	2.904 (5)	145
N4—H4A···O5^iii^	0.86	2.04	2.878 (5)	166
O9—H1W···O4^iv^	0.85	1.89	2.730 (5)	170
O9—H2W···O7^v^	0.85	2.06	2.834 (5)	150
O10—H4W···O8^vi^	0.85	2.22	2.958 (5)	145

Symmetry codes: (i) *x*, *y*, *z*+1; (ii) −*x*+1/4, *y*+1/4, *z*−3/4; (iii) −*x*+1/4, *y*+1/4, *z*+1/4; (iv) *x*−1/4, −*y*+1/4, *z*+3/4; (v) *x*, *y*, *z*−1; (vi) *x*+1/4, −*y*+1/4, *z*−3/4.
